# Impaired mixed emotion processing in the right ventrolateral prefrontal cortex in schizophrenia: an fMRI study

**DOI:** 10.1186/s12888-017-1558-x

**Published:** 2017-12-08

**Authors:** Ádám György Szabó, Kinga Farkas, Csilla Marosi, Lajos R. Kozák, Gábor Rudas, János Réthelyi, Gábor Csukly

**Affiliations:** 1 Department of Psychiatry and Psychotherapy, Semmelweis University, Balassa 6, Budapest, 1083 Hungary; 2 MR Research Center, Semmelweis University, Budapest, Hungary

**Keywords:** Emotion processing, Insula, Schizophrenia, Vlpfc, Prefrontal, Temporal

## Abstract

**Background:**

Schizophrenia has a negative effect on the activity of the temporal and prefrontal cortices in the processing of emotional facial expressions. However no previous research focused on the evaluation of mixed emotions in schizophrenia, albeit they are frequently expressed in everyday situations and negative emotions are frequently expressed by mixed facial expressions.

**Methods:**

Altogether 37 subjects, 19 patients with schizophrenia and 18 healthy control subjects were enrolled in the study. The two study groups did not differ in age and education. The stimulus set consisted of 10 fearful (100%), 10 happy (100%), 10 mixed fear (70% fear and 30% happy) and 10 mixed happy facial expressions. During the fMRI acquisition pictures were presented in a randomized order and subjects had to categorize expressions by button press.

**Results:**

A decreased activation was found in the patient group during fear, mixed fear and mixed happy processing in the right ventrolateral prefrontal cortex (VLPFC) and the right anterior insula (RAI) at voxel and cluster level after familywise error correction. No difference was found between study groups in activations to happy facial condition. Patients with schizophrenia did not show a differential activation between mixed happy and happy facial expression similar to controls in the right dorsolateral prefrontal cortex (DLPFC).

**Conclusions:**

Patients with schizophrenia showed decreased functioning in right prefrontal regions responsible for salience signaling and valence evaluation during emotion recognition. Our results indicate that fear and mixed happy/fear processing are impaired in schizophrenia, while happy facial expression processing is relatively intact.

## Background

Schizophrenia is characterized with neurocognitive and social cognitive impairments, both of which contribute to decreased psychosocial functioning [1]. Social cognition consists of three major areas: attributional style, theory of mind and emotion recognition [2]. In the present investigation the focus was on emotion processing and recognition from facial displays.

Brain regions involved in emotion processing are the amygdala, the basal ganglia, the lateral and medial parietal cortex, the medial temporal lobe, the lateral temporal cortex, the dorsal and rostral anterior cingulate cortex, the anterior insular cortex, and the prefrontal cortex [3, 4]. The amygdala is primarily responsible for the cognitive representation of fear [5]. The temporal-parietal structures are activated in the process of face and facial expression detection [6]. The ventromedial prefrontal cortex (VMPFC) and the dorsolateral prefrontal cortex (DLPFC) are involved in the regulation of cognitive functions linked to emotions such as attention, while the ventrolateral prefrontal cortex (VLPFC) signals emotion salience and the need to regulate [7, 8].The right VLPFC is also implicated in the integration of viscerosensory information with affective signals between the bilateral anterior VLPFCs and the bilateral amygdale [9] and activated in situations with decision uncertainty [10]. The anterior insula and anterior cingulate cortex are involved in emotional tasks with cognitive demand [4].

Multiple previous studies have shown that schizophrenia has an effect on the functioning of the temporal [11] and prefrontal cortices [12], including the detection, evaluation and reappraisal of facial emotions. A recent study also revealed that the neural networks of frontolimbic regions are impaired in adolescents at high risk for schizophrenia [13].

While several previous investigations examined the recognition of basic emotions in schizophrenia [14], only a few studied the processing of different emotion intensities, while no previous research focused on the evaluation of mixed emotions, however they are requently expressed in everyday situations. Furthermore in many cases negative emotions such as fear are expressed by mixed facial expression (e.g. a mixture of a smile and a fearful facial display). Therefore using mixed emotions can be more ecological valid. Hence in the present investigation facial displays of mixed positive and negative emotions were also presented to patients with schizophrenia and healthy subjects. We hypothesized that the processing of these more complex emotions are more severely impaired in schizophrenia, and therefore a better differentiation of patients from controls can be achieved based on activation patterns. Specifically decreased activations were expected in regions involved in facial emotion processing in the patient group, especially in prefrontal regions, where previous investigation found activation in task with high cognitive demand linked to emotion processing such the anterior insula [4] and also in regions linked to salience signaling such as the ventrolateral prefrontal cortex [15].

## Methods

### Subjects

Altogether 19 patients with schizophrenia and 18 healthy control subjects were enrolled in the study. All participants underwent a series of psychological tests, EEG (electroencephalographic) examinations and finally a set of functional MRI (magnetic resonance imaging) tasks. The two study groups did not differ in age and education (Table 1). All participants were right-handed with the exception of one left-handed and one mixed handed patient and two left-handed healthy controls. All participants had normal or corrected-to-normal vision. Selection criteria for all participants were no history of any CNS (central nervous system) disease, mental retardation, epileptic seizure, substance dependence or substance abuse in the past 3 months, no history of head injury with loss of consciousness more than 10 minutes. For healthy controls further exclusion criteria were any psychiatric disorder and a global severity index of >114 on the Symptom Checklist–90–R, [16] according to a Hungarian population sample [17], in order to exclude subjects with high risk for psychiatric disorders.

Patients were recruited from the Department of Psychiatry and Psychotherapy, Semmelweis University, Budapest, Hungary. All patients met the criteria for schizophrenia based on the Structured Clinical Interview for Diagnostic and Statistical Manual of Mental Disorders, 4^th^Edition (DSM-IV)(American Psychiatry Association 1994). The Positive and Negative Syndrome Scale (PANSS) were evaluated by a trained psychiatrist [18]. At the time of testing all patients took antipsychotic medication, the mean Chlorpromazine equivalent dose (Gardner et al. 2010) was 716 mg/day (SD = 337), while 10 patients took Benzodiazepines (BZD), and the mean Clonazepam equivalent dose (CPZ) was 1.6 mg/day (SD = 1.1 mg). Psychosocial functioning was measured by the Personal and Social Performance Scale (PSP) [19]. Demographic information for both study groups and the clinical characteristics of the patient group are presented in Table 1 **.**

**Table 1:** Demographic information for both study groups and clinical characteristics of the schizophrenia group

	Schizophrenia Group (*n* = 19) Mean (SD)	Control Group (*n* = 18) Mean (SD)	statistics	*p* value
Gender (male/female)	12 / 6	11/8	Chi^2^ = 0.3	n.s.
Age	37.4 (8.4)	37.6 (10.9)	*t* = 0.1	n.s.
Education^a^	2/5/6/6	1/2/7/8	Fisher’s exact test	n.s.
Illness duration (years)	12.2 (6.3)	–		
In−/Outpatients	9 / 10	–		
CPZ equivalent dose	716 mg (337)	–		
PANSS total score	77.7 (18.5)	–		
PANSS positive subscore	17.6 (6.7)	–		
PANSS negative subscore	20.3 (6.8)	–		
PANSS general subscore	39.8 (9.1)	–		
PSP	59.5 (16.1)	–		

^a^1 = elementary school; 2 = high school; 3 = vocational school; 4 = college/university

(CPZ = chlorpromazine equivalent dose; PANSS = Positive and Negative Symptoms Scale; PSP = Personal and Social Performance Scale)

### Procedures

#### Stimuli

In order to generate mixed emotional facial expressions morphed faces from the Ekman database [20] were used. These morphed faces were tested in a different group of healthy subjects (*n* = 20), who had not participated in the MRI study. Subjects had to select the faces which expressed the mixture of the two emotions the best, but dominantly happy or fear respectively. The ones with 30% and 70% mixture of the emotions were selected for the final task as mixed emotions. We refer to these mixed emotions by the 70% component: mixed fear is 70% fear and 30% happy, while mixed happy is 70% happy and 30% fear. The stimulus set consisted of 10 fearful (100%) facial expression, 10 happy (100%) faces and 10 mixed fear and 10 mixed happy faces.

**Fig. 1:**
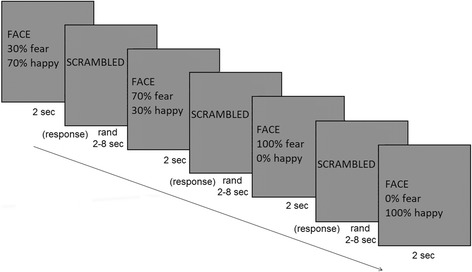
Schematic illustration of the experimental paradigm. Pictures of happy (100%), fear (100%), mixed happy (70% happy and 30% fear) and mixed fear (70% fear and 30% happy) facial expression (http://www.paulekman.com) were presented for 2 s in a randomized order, the inter-stimulus baseline was a distorted (scrambled) picture, which was a Fourier transformation of all the original images. The inter-stimulus interval was randomized between 2 and 10 s. The subjects had to respond by a button press whether they see a happy or a fearful facial expression

All pictures were presented for 2 s in a randomized order, the inter-stimulus baseline was a distorted (scrambled) picture, which was a Fourier transformation of all the original images (Fig. 1). The inter-stimulus interval was randomized between 2 and 10 s. The subjects had to respond by a button press whether they had seen a happy or a fearful facial expression. The viewing distance was 60 cm. Happy facial expression was selected as counterparts of fearful faces, since the recognition of happiness was found to be mildly affected compared to impaired fear processing in patient with schizophrenia according to previous studies [21], while the processing of neutral facial expressions were found to be severely impaired in schizophrenia [22–24]. We decided to exclude neutral facial displays from the paradigm due to the following reasons: using three response buttons may overcomplicate the task and the increasing number of stimuli would have made the task too long, both of which might have raised attentional issues and might have biased the results, especially in the patient group.

#### Image acquisition

The described MRI procedure took 30 min and were done at the MR Research Center, Semmelweis University on a 3 Tesla Philips Achieva whole body clinical MRI scanner (Philips Medical Systems, Best, The Netherlands) equipped with an 8-channel SENSE head-coil. The high resolution, whole brain anatomical images were obtained using a T1 weighted 3 dimensional spoiled gradient echo (T1 W 3D TFE) sequence. 180 contiguous slices were acquired from each subject with the following imaging parameters: TR (time resolution) = 9.7 ms; TE (echo time) = 4.6 ms; flip angle = 8°; FOV (Field-of view) of 240 mm × 240 mm; voxel size of 1.0 × 1.0 × 1.0 mm. Functional images were acquired using a T2* weighted gradient echo echo-planar imaging sequence with the following parameters: TR = 2.0 ms; TE = 30 ms; flip angle = 70°, FOV of 240 mm × 240 mm; voxel size of 3.0 × 3.0 × 4.0 mm; maximum number of slices = 36. NordicNeuroLab SyncBox and ResponseGrip (NordicNeuroLab, Bergen, Norway) were used to record subject responses during tasks. Stimuli were presented in Psychtoolbox (http://psychtoolbox.org/), a Matlab R2012A (Mathworks, Natick, MA, USA) toolbox used for visual stimulus presentation. In During the fMRI experiment visual stimuli were projected onto a translucent screen located at the back of the scanner bore using an Panasonic Panasonic PT-D3500E DLP projector (Matsushita Electric Industrial Co., Osaka, Japan) at a refresh rate of 75 Hz. Stimuli were viewed from inside the magnet bore by through a mirror system attached to the head coil, the viewing distance was 58 cm. Head motion was minimized using foam padding.

#### MRI preprocessing and statistical analysis

Image preprocessing was performed using Statistical Parametric Mapping (SPM12, Wellcome Department of Cognitive Neurology, London, UK) under Matlab 2012 (Mathworks, Natick, MA, USA). Echo-planar imaging (EPI) data of each subject were preprocessed, which included slice timing, realignment and normalization into a standard template (Montreal Neurological Institute, MNI). A motions threshold of 3mms were used (equals to voxel size), any subjects moved beyond this threshold were excluded. Based on these criteria altogether four subjects were excluded from the study. Normalized images were finally smoothed in space with an 8 mm full-width at half-maximum (FWHM) 3D isotropic Gaussian kernel. Images were finally filtered with a high-pass filter of 128 s to remove low frequency drifts. After preprocessing, statistical analyses for each individual subject was performed based on fixed-effects general linear models (GLM). Individual events were modeled as single delta (aka stick) functions at each stimulus onset and convolved with the canonical hemodynamical response function (HRF). In the 1st level analysis the four conditions (happy, fear, mixed happy, mixed fear) were modelled, while the 24-motion parameters were included as nuisance regressors (the motion parameters, their first derivatives, the squared motion parameters, and the squared derivatives). Group level analyses was performed by Statistical Non-Parametric Mapping (SNPM version 13) (http://warwick.ac.uk/snpm), a permutation based approach [25]. In the second level analysis both voxel-wise and cluster-wise results were calculated with 10.000 permutations (pseudo-T values were calculated), in the latter case a cluster defining threshold (CDT) of *p* < 0.001 was used in SNPM to determine significant activations. Age and gender were included as covariates in the analysis. The results of the 2nd level analysis are presented in Table 2 including corresponding anatomical regions and Brodmann areas detected using xjView toolbox (http://www.alivelearn.net/xjview).

**Table 2:** Between group differences in brain activations

Condition/Anatomical region	Brodmann areas (BA)	Peak MNI coordinates			Voxel level	Cluster level	
		X	Y	Z	*p* value (FWE-corr.)	cluster size (voxels/cm^3^)	*p* value (FWE-corr.)
Fear (100% Fear)							
Right Middle Frontal Gyrus (RMFG)	10	42	50	2	0.01	181/6.5	0.006
Right Anterior Insula (RAI), Right Ventrolateral Prefrontal Cortex (VLPFC)	13, 47	45	20	−6	0.01	166/6.0	0.006
Right and Left Medial Frontal Gyrus, Dorsomedial Prefrontal Cortex (DMPFC)	8, 9	3	35	42	0.03	142/5.1	0.008
Mixed fear (70% Fear / 30% Happy)							
Right Anterior Insula (RAI), Right Ventrolateral Prefrontal Cortex (VLPFC)	13, 47	45	20	−6	0.003	218/7.8	0.002
Mixed happy (70% Happy/30% Fear)							
Right Anterior Insula (RAI), Right Ventrolateral Prefrontal Cortex (VLPFC)	13, 47	33	35	−10	0.03	171/6.2	0.004
Right Middle Occipital Gyrus (RMOG)	18	39	−85	−6	ns.	54/1.9	0.04

Additionally correlational analyses were performed between clinical variables (PANSS, PSP, AP and BZD dose) and the activations in the clusters/regions where group differences were found. Correlational analyses were performed in SAS 9.3 (SAS Institute Inc., Cary, NC, USA). Further ROIs selected from the AAL (Automated Anatomical Labeling) brain atlas [26] corresponding to emotion processing [3] were created and extracted by Marsbar [27]. Correlational analyses were performed for these 14 ROIs (Left and Right Amygdala, Fusiform gyrus, Superior/Middle/Inferior Temporal gyrus and Superior/Middle Temporal Pole) with the same clinical variables as listed above.

Cortical reconstruction and volumetric segmentation were performed with the Freesurfer 5.3 image analysis suite, which is documented and freely available for download online (http://surfer.nmr.mgh.harvard.edu/), and the results contain information about each vertex of the created mesh. The technical details of these procedures are described in prior publications; we made no changes to this pipeline. Segmentations and cortical models were checked and corrected manually on each subject. We used FreeSurfer’s QDEC (Query, Design, Estimate, Contrast) interface to perform group averaging and generate the statistical maps from the cortical morphometric data produced by FreeSurfer stream. General Linear Model (GLM) was computed vertex-by-vertex for analysis of surface area and cortical thickness. The cortical volumes of the schizophrenic patient group was compared with healthy controls, accounting for the effects of age and gender. From the different methods that QDEC provides for automatic design matrix creation, DODS (different offset, different slope) was used. The results were visualised by overlaying the significant areas onto the cortical surfaces, maps were smoothed using a 10 mm full width at half maximum Gaussian kernel. Multiple comparisons were corrected with a Monte Carlo Simulation using a *p*-value set at <0.05.

## Results

### Differentiation between study groups based on BOLD activations

**Fig. 2:**
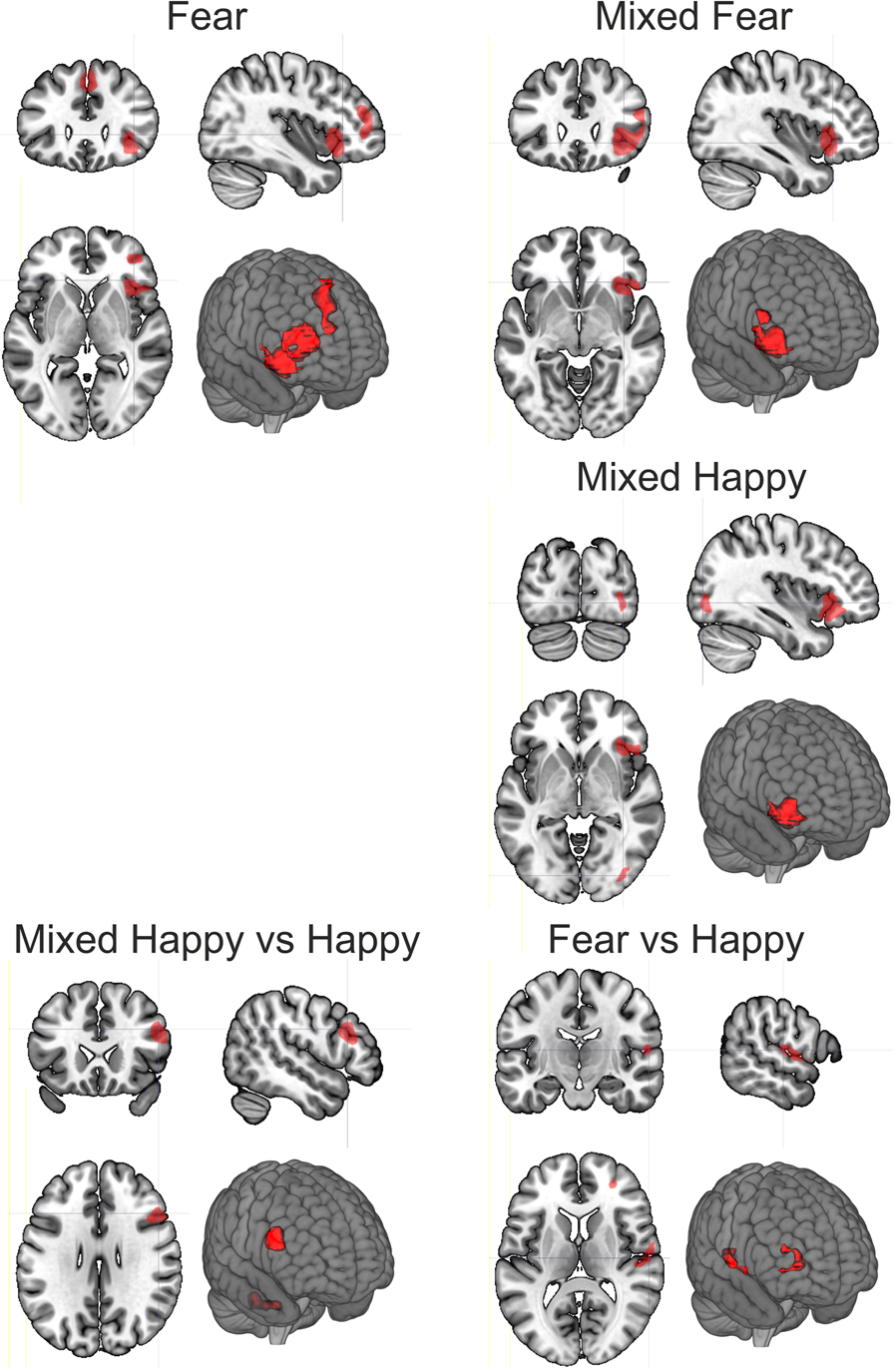
Between group differences in brain activation to fear, mixed fear, and mixed happy stimuli respectively.1 Fear: Right Middle Frontal Gyrus (RMFG), Right Anterior Insula (RAI), Right Ventrolateral Prefrontal Cortex (VLPFC), Right and Left Medial Frontal Gyrus, Dorsomedial Prefrontal Cortex (DMPFC). 2 Mixed Fear: Right Anterior Insula (RAI), Right Ventrolateral Prefrontal Cortex (VLPFC). 3 (Happy: no between group difference). 4 Mixed Happy: Right Anterior Insula (RAI), Right Ventrolateral Prefrontal Cortex (VLPFC), Right Middle Occipital Gyrus (RMOG) The clusters corresponding to the interactions between stimulus condition and study group are also presented. 1 Fear vs. Happy: Right Superior Temporal Gyrus (RSTG), Right Rolandic Operculum (RRO), Right Middle Frontal Gyrus (RMFG). 2 Mixed Happy vs. Happy:, Vermis of the Cerebellum Right Middle and Inferior Frontal Gyrus, Right Dorsolateral Prefrontal Cortex (DLPFC). Results are based on permutation based statistics (*n* = 10,000) from Statistical non-Parametric Mapping (SnPM).

Decreased activation was found in the patient group during fear (Fig. 2/A) and mixed fear (Fig. 2/B) processing in clusters corresponding to the right VLPFC (Brodmann Area 47 = BA47), the right anterior insula (RAI/BA13), and the right dorsomedial prefrontal cortex (DMPFC/BA9) at voxel and cluster level. The results of the analyses are summarized in Table 2.

A significant decrease at voxel and cluster level activation was also found in the right VLPFC and right anterior insula (RAI), and at cluster level in the right middle occipital gyrus (BA18/associative visual cortex) in patients when viewing mixed happy faces (Table 2 **;** Fig. 2/B). No difference was found between study groups to happy faces in any regions.

**Fig. 3:**
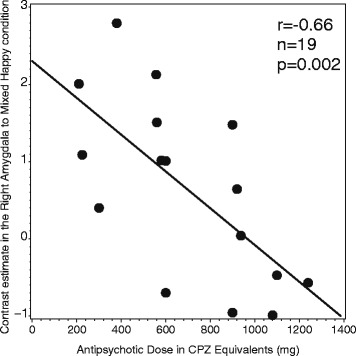
The correlation of the contrast estimate in the Right Amygdala to Mixed Happy condition and Antipsychotic Dose in CPZ Equivalents in patients with schizophrenia

Activations to all four emotion conditions in the right amygdala correlated negatively with antipsychotic dose, which was the strongest for mixed happy condition (mixed happy: *r* = −0.66, *n* = 19, *p* = 0.002; happy: *r* = −0.55, *n* = 19, *p* = 0.02; mixed fear: *r* = −0.47, n = 19, *p* = 0.04; fear: *r* = −0.48, *n* = 19, p = 0.04) (Fig. 3). Correlations of brain activations with psychotic symptom severity, psychosocial functioning and BZD doses did not reach significance in any regions.

### Interactions between study group and condition

**Fig. 4:**
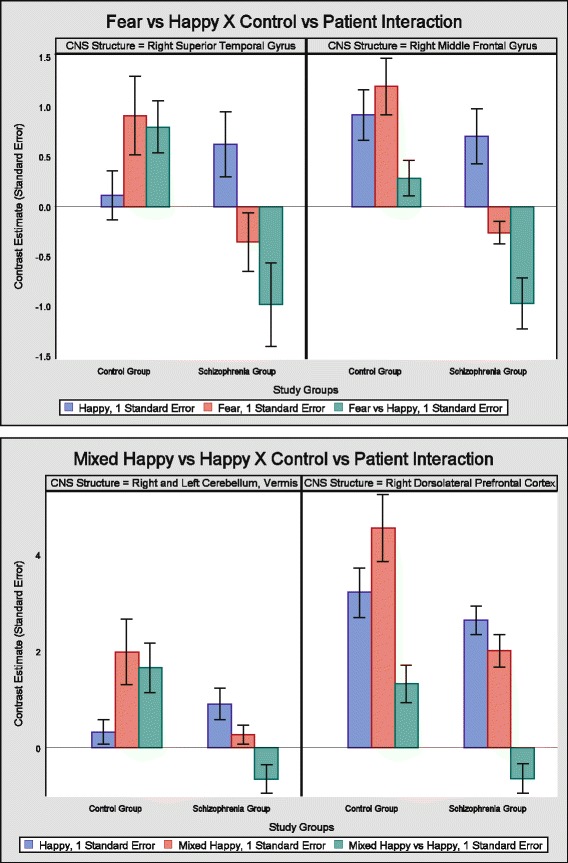
On the top pane the study group x (Fear vs Happy) interaction is presented, while on the bottom pane the study group x (Mixed Happy vs Happy) interaction is presented. Blue bars represent contrast estimates to happy conditions, red bars represent contrast estimates to fear (top pane) and to mixed happy (bottom pane) conditions, while green bars represent the difference in contrast estimates between the former two stimuli

A study group X condition (Fear vs Happy) interaction was found significant (FWE corrected *p* = 0.03) in a 62 voxel cluster in the right parietal operculum (RPO) and right superior temporal gyrus (RSTG/BA22) and also in the right middle frontal gyrus (RMFG/BA10) (FWE corrected *p* = 0.04) in a 50 voxel cluster (Table 3 **;** Fig. 2/C). Further analyzing average activations in these clusters (Fig. 4), increased activations to fear compared to happy facial expressions were found in controls in RPO and RSTG (*p* = 0.004), and in RMFG (*p* = 0.04), while there were increased activations to happy compared to fearful faces in patients in RPO and RSTG (*p* = 0.006), and in RMFG (*p* < 0.0001).

**Table 3:** Interaction between study group and stimulus condition in bran activations

Condition/Anatomical region	Brodmann areas (BA)	Peak MNI coordinates			Voxel level	Cluster level	
		X	Y	Z	*p* value (FWE-corr.)	cluster size (voxels/cm^3^)	*p* value (FWE-corr.)
(Fear vs Happy) X (Control vs Patient)							
Right Superior Temporal Gyrus (RSTG), Right Rolandic Operculum (RRO)	22	60	−4	6	ns.	62/2.2	0.03
Right Middle Frontal Gyrus (RMFG)	10	30	47	6	ns.	50/1.8	0.04
(Mixed Happy vs Happy) X (Control vs Patient)							
Right and Left Cerebellum, Vermis	NA	3	−76	−38	< 0.05	94/3.4	0.01
Right Middle and Inferior Frontal Gyrus, Right Dorsolateral Prefrontal Cortex (DLPFC)	9, 46	51	23	22	0.06	80/2.9	0.02

Also the study group X condition (Mixed Happy vs Happy) interaction was found significant (FWE corrected *p* = 0.01 (cluster level) *p* < 0.05 (peak voxel)) in a 94 voxel cluster in the vermis of the Cerebellum (VC) and in the right dorsolateral prefrontal cortex (DLPFC/BA46) (FWE corrected *p* = 0.02) in a 80 voxel cluster (Table 3 **;** Fig. 2/C). Further analyzing average activations in these clusters (Fig. 4), increased activations to mixed happy compared to happy facial expressions were found in controls both in VC and (*p* < 0.0001), and in DLPFC (*p* < 0.0001), while there were no significant between condition differences in the patient groups in any of these regions.

### Behavioral results

#### Recognition performance

The study groups did not differ significantly in the recognition of happy, mixed happy, and fearful facial expressions (happy: Control subjects (Cntrl) = 92% (SD = 17%), Patients with Schizophrenia (Sch) = 98% (SD = 4%), *t* = 1.2, *p* = 0.2; mixed happy: Cntrl = 86% (SD = 17%), Sch = 90% (SD = 15%), *t* = 0.9, *p* = 0.4; fear: Cntrl = 99% (SD = 3%), Sch = 99% (SD = 3%), *t* = 0.2, *p* = 0.9), while patients recognized mixed fear emotions as fear at a significantly lower rate compared to controls (mixed fear: Cntrl = 90% (SD = 9%), Sch = 84% (SD = 11%), *t* = 2.4, *p* = 0.02, Effect Size = 0.6 in terms of Cohen’s d).

#### Reaction times

The two groups did not differ in reaction times (overall: Cntrl = 1.13 s (SD = 0.17 s), Sch = 1.21 s (SD = 0.23 s), *t* = −1.2, *p* = 0.24; happy: Cntrl = 1.07 s (SD = 0.19 s), Sch = 1.24 s (SD = 0.32 s), *t* = −1.9, *p* = 0.06; mixed happy: Cntrl = 1.26 s (SD = 0.23 s), Sch = 1.39 s (SD = 0.33 s), *t* = −1.4, *p* = 0.18; fear: Cntrl = 1.18 s (SD = 0.24 s), Sch = 1.47 s (SD = 0.36 s), *t* = −2.1, *p* = 0.054; mixed fear: Cntrl = 1.60s (SD = 0.33 s), Sch = 1.54 s (SD = 0.30s), *t* = 0.5, *p* = 0.61).

#### Volumetric comparison of patients and controls

The primary purpose of this analysis was to check if functional activation differences between the groups can be explained by volumetric differences. After Familywise Error Correction (FEW) no significant structural differences were found between the study groups.

## Discussion

In this study we examined emotion processing in a group of patients with schizophrenia and in healthy controls. The study groups were matched by age, gender, and education. Patients showed a decreased activation in the right anterior insula (RAI), in the right VLPFC, in the right DMPFC during fear, mixed fear, and mixed happy facial processing and showed poorer emotion recognition performance in fear detection relative to controls. In patients on higher doses of APs decreased activations to all emotional displays were detected in the right anterior amygdala, which correlation was the strongest for mixed happy faces.

Subjects were presented with facial displays of happiness, fear, and the mixture of these emotions as described in the methods section. From previous research we know that VLPFC activation is related to emotion intensity and reappraisal [7, 28], and signals the salience of the stimulus [8]. Moreover, emotional tasks with cognitive demand involves primarily the anterior cingulate and insula [4]. Decreased activation was detected in the right anterior insula (RAI), and in the VLPFC region to fear, mixed fear, and mixed happy conditions in patients relative to controls. Previous investigations showed that VLPFC plays a key role in emotion regulation and reappraisal through cortical-subcortical pathways especially to aversive stimuli [29]. It is also found that VLPFC activity correlated with reduced negative emotional experience during cognitive reappraisal of aversive images [30]. Taken these results together with our findings raised the possibility that salience signaling and perhaps also emotion regulation linked to negative emotion processing are impaired in schizophrenia, which is in line with previous studies examining emotion processing in schizophrenia that found decreased activation in the VLPFC relative to controls [12, 15, 31].

Patients showed decreased activation relative to controls in the right middle occipital gyrus (RMOG/BA18) to facial conditions of mixed happy facial stimuli. The RMOG/BA18 is part of the ventral stream of visual processing for which preferential processing of affective stimuli has previously been demonstrated [32]. Previous studies found increased activation in the RMOG/BA18 to fearful faces compared to happy stimuli during facial emotion processing [32], furthermore a recent study found that this region is under control by the anterior cingulate cortex during facial emotion processing [33]. Our finding is in line with previous results showing an early visual processing impairment in emotional facial processing in schizophrenia [34–36]. Furthermore patients with schizophrenia showed decreased activations in the right middle frontal cortex (RMFG/BA10) and in the dorsomedial prefrontal cortex (DMPFC) to fearful facial stimuli. According to previous investigations decreased DMPFC activity in response to emotion facial cues may reflect less cognitive control involved in decoding and/or regulating negative emotions [37], which was also observed in chronically violent men [38], and in postpartum depression [39]. A study examining the theory of mind impairments in subjects with autistic spectrum disorder found decreased activity in the DMPFC during inferring other person’s social emotions in a false belief task [40].

Patients on higher doses of APs showed decreased activation in the right amygdala to all emotional faces with the strongest correlation for mixed happy condition. Little is known so far on the effects of AP treatment on emotion processing in schizophrenia. In a previous study Sachs et al. [41] reported no difference in amygdala activation between patients treated with atypical APs and healthy subjects. In another study 12 previously drug-free/naive patients with schizophrenia were treated with olanzapine for 8 weeks and underwent two fMRI scans after 4 and 8 weeks of treatment during implicit and explicit emotional processing. Activity in left amygdala was greater in patients than in controls at the first scan during both explicit and implicit processing, while it was lower in patients at the second relative to the first scan [42]. However none of the previous studies reported any dose related effect of APs on the activity of brain regions involved in emotion processing. Contrary to previous investigations no between group differences were found in the amygdala activation to fearful faces. A possible explanation to that is that fearful faces in the present study were not as strong fear evoking stimuli as the scenarios depicted in the International Affective Picture Set (IAPS) [43].

Previous studies found that the superior temporal gyrus and the insular cortex have been involved in the perception of emotions in facial stimuli [5, 44], while the BA10 region is mainly activated during multi-tasking and theory of mind tasks [45]. In the present study an increased activation to fearful faces relative to happy faces were detected in the right superior temporal gyrus and in the right middle frontal gyrus (BA10) in healthy subjects, while the opposite difference (happy > fear) was found in patients. In a previous investigation, similarly to our results, an increased activation to fearful stimuli relative to safe stimuli was detected in the insular cortex in healthy subjects [44].

The activation of the DLPFC is related to the regulation of cognitive processes linked to emotion processing such as attention [7, 28]. A decreased activation difference between mixed happy faces and happy faces in patients relative to controls were found in the cerebellum (vermis) and in the right DLPFC. Controls showed an increased activation to mixed happy faces relative to happy faces in these regions, while similar between emotion differences were not found in patients. These findings are in line with previous investigations showing that during emotion processing [46] and social cognition tasks [47] patients with schizophrenia showed decreased activations in the DLPFC and in the cerebellum during face processing [48].

Structural impairments in patients may explain the functional differences, since some previous investigations found structural differences in patients with schizophrenia relative to healthy controls in temporal [49] and frontal regions. However no similar volumetric differences were found in the present study.

A major limitation of the present study was that no neutral facial expressions were used. The reason was that we intended keep the paradigm as short as possible to avoid attention related confounds. A further limitation was that patients were at antipsychotic medication, which may bias the activations, however there were no correlations between antipsychotic doses and BOLD activations in the regions where the between group differences were found. Study groups were matched by age, gender, and education, however this approach has the drawback that it does not take into consideration that patients with schizophrenia experience a significant reduction in educational attainment relative to what we be predicted had they not received such a diagnosis. Due to a technical malfunction of the input device several responses were lost, which made it impossible to include responses into the 1st level model. This may lead to confounds related to the motor activation needed to produce a button press, however no between study group differences were detected in motor areas or supplementary motor regions, which makes it unlikely that this was a major confound.

## Conclusions

Patients with schizophrenia showed decreased activation in the right anterior insula (RAI) and in the right VLPFC during facial emotion processing. Whereas the former region is activated in emotional tasks with cognitive demand, the latter is responsible for salience signaling [8]. These impaired process were detected in response to facial expressions containing more or less fear with or without happiness, while no similar impairment was detected in the processing of happy facial expressions without fear. Therefore we can conclude that fear and mixed happy/fear processing are impaired in schizophrenia, while happy facial expression processing is relatively intact. Furthermore patients with schizophrenia did not show a differential activation between mixed happy and happy facial expressions similar to controls in the right DLPFC, a region related to the regulation of cognitive processes linked to emotions such as attention, and valence evaluation [8]. In sum patients with schizophrenia showed decreased functioning in right prefrontal regions responsible for salience signaling and valence evaluation during emotion recognition.

## Abbreviations

AAL: Automated Anatomical Labeling
AP: Antipsychotic
BA: Brodmann Area
BOLD: Blood Oxygen Level Dependent
BZD: Benzodiazepine
CDT: cluster defining threshold
CNS: Central Nervous System
Cntrl: Control subjects
CPZ: Chlorpromazine Equivalent Dose
DLPFC: Dorsolateral Prefrontal Cortex
DLPFC: Dorsolateral Prefrontal Cortex
DMPFC: Dorsomedial Prefrontal Cortex
DODS: Different Offset, Different Slope
DSM-IV: Diagnostic and Statistical Manual of Mental Disorders, 4^th^Edition
EEG: Electroencephalogram
EPI: Echo-planar imaging
FEW: Familywise Error Correction
fMRI: Functional Magnetic Resonance Imaging
FOV: Field-of-view
FWHM: Full width at half maximum
GLM: General Linear Model
HRF: Hemodynamical Response Function
IAPS: International Affective Picture Set
MNI: Montreal Neurological Institute
MRI: Magnetic Resonance Imaging
PANSS: Positive and Negative Symptoms Scale
PSP: Personal and Social Performance Scale
QDEC: Query, Design, Estimate, Contrast
RAI: Right Anterior Insula
RMFG: Right Middle Frontal Cortex
RMOG: Right Middle Occipital Gyrus
ROI: Region of Interest
RPO: Right Parietal Operculum
RRO: Right Rolandic Operculum
RSTG: Right Superior Temporal Gyrus
Sch: Patients with Schizophrenia
SD: Standard Deviation
SNPM: Statistical non-Parametric Mapping
T1 W 3D TFE: T1 weighted 3 dimensional spoiled gradient echo
TE: Echo Time (fMRI)
TR: Temporal Resolution (fMRI sampling time)
VC: Vermis of the Cerebellum
VLPFC: Ventrolateral Prefrontal Cortex
VMPFC: Ventromedial Prefrontal Cortex
